# Transgene Excision Has No Impact on In Vivo Integration of Human iPS Derived Neural Precursors

**DOI:** 10.1371/journal.pone.0024687

**Published:** 2011-09-22

**Authors:** Tamara Major, Jayanthi Menon, Gordon Auyeung, Frank Soldner, Dirk Hockemeyer, Rudolf Jaenisch, Viviane Tabar

**Affiliations:** 1 Center for Stem Cell Biology and Department of Neurosurgery, Memorial Sloan Kettering Cancer Center, New York, New York, United States of America; 2 The Whitehead Institute, Massachusetts Institute of Technology, Cambridge, Massachusetts, United States of America; 3 Department of Biology, Massachusetts Institute of Technology, Cambridge, Massachusetts, United States of America; Duke University Medical Center, United States of America

## Abstract

The derivation of induced human pluripotent stem cells (hiPS) has generated significant enthusiasm particularly for the prospects of cell-based therapy. But there are concerns about the suitability of iPS cells for in vivo applications due in part to the introduction of potentially oncogenic transcription factors via viral vectors. Recently developed lentiviral vectors allow the excision of viral reprogramming factors and the development of transgene-free iPS lines. However it is unclear if reprogramming strategy has an impact on the differentiation potential and the in vivo behavior of hiPS progeny. Here we subject viral factor-free, c-myc-free and conventionally reprogrammed four-factor human iPS lines to a further challenge, by analyzing their differentiation potential along the 3 neural lineages and over extended periods of time in vitro, as well as by interrogating their ability to respond to local environmental cues by grafting into the striatum. We demonstrate similar and efficient differentiation into neurons, astrocytes and oligodendrocytes among all hiPS and human ES line controls. Upon intracranial grafting in the normal rat (Sprague Dawley), precursors derived from all hiPS lines exhibited good survival and response to environmental cues by integrating into the subventricular zone, acquiring phenotypes typical of type A, B or C cells and migrating along the rostral migratory stream into the olfactory bulb. There was no teratoma or other tumor formation 12 weeks after grafting in any of the 26 animals used in the study. Thus neither factor excision nor persistence of c-myc impact the behavior of hiPS lines in vivo.

## Introduction

The derivation of human iPS cells [1], [2] is a major advance for biomedical research including cell-based therapies. However there have been several concerns about the suitability of iPS cells for in vivo applications. Those include a risk of insertional mutagenesis or point mutations in protein-coding regions [3] following the use of integrating vectors, aberrations in epigenetic reconfiguration [4] or incomplete silencing of transgenes with reactivation of potentially oncogenic factors such as c-myc [5]. In addition, questions pertaining to the differentiation potential and in vivo behavior of iPS cells, compared to human ES cells remain unresolved. While iPS cells closely mimic the properties of ES cells, recent studies in the mouse have shown differences in epigenetic state [6], [7] related to cell of origin. Other investigations using the reprogrammable mouse system showed differences in the expression of specific imprinted genes [8] that were correlated with the ability of the cells to contribute to chimeric mice in vivo. A number of studies also showed differences in gene expression between iPS and ES cells, though some of those alterations might be due to residual transgene expression in the resulting iPS cells [9]. A recently developed doxycycline-inducible lentiviral vector system enables transgene excision by Cre-recombinase resulting in transgene-free iPS lines [9]. The lines and specific clones used here have been published and fully characterized by Soldner et al. [9], including an assessment of their pluripotency via factor expression and teratoma formation; their karyotype was also shown to be normal. Interestingly, gene expression studies demonstrated significant differences between excised versus non-excised iPS cell lines with the excised clones more closely matching the gene expression profiles of ESC lines[9]. In this work, we focus on the in vivo behavior of iPS progeny, taking advantage of the availability of iPS lines that are derived from the same fibroblast source and that differ by the ability of actively excising the reprogramming transgenes.

We subjected iPS clones derived, via several reprogramming strategies, from a single fibroblast line (+/− c-myc, excised or non-excised transgenes), to neural induction and differentiation along neuronal, astrocytic and oligodendroglial lineages. Comparisons were established side-by-side with a well-characterized ES line (H9, WA-09). We also asked whether the persistence of viral factor expression could impact more complex interactions of human iPS progeny with the host brain microenvironment in vivo. Using a previously established transplantation paradigm [10] we compared the potential of human neural precursors derived from excised versus non-excised iPS or from control ES cell lines for in vivo integration into the adult rat SVZ and the ability to contribute human cells to the rostral migratory stream and olfactory bulb. Of particular interest was the question of whether residual expression of c-myc might induce excessive proliferation or even promote neoplastic transformation in vivo. Using viral transgene factor-free, c-myc-free and conventionally reprogrammed four-factor human iPS lines, we observed a comparable differentiation potential along the 3 major neural lineages in vitro, including the derivation of oligodendrocyte and astrocyte lineages. Similarly, the in vivo study demonstrated that neural precursors are capable of responding to local environmental cues by integration into the adult brain subventricular zone (SVZ) independently of transgene excision. These data suggest that carefully validated iPS cell clones are suitable for in vitro and in vivo iPS cell differentiation and for transplantation studies independent of transgene excision.

## Results

### Neural induction in iPS cells

Induced pluripotent stem cell (hiPSC) lines were generated from a single source of dermal fibroblasts (AG20442) as described previously[9]. In brief, the lines used in the current study include PDB^4F^-2 and PDB^3F^-5 which were derived using doxycycline (DOX)-inducible lentiviruses carrying *OCT4*, *SOX2*, *KLF4* with (4F) or without c-myc (3F). An additional pair of hiPS lines represents a parental line reprogrammed using 3 non-excised factors (no c-myc; PDB^2lox^-21) and its derivative, subjected to Cre-recombinase mediated excision of reprogramming factors (PDB^1lox^-21-12). Absence of residual factors in the excised lines was confirmed by Southern blot. The human ES line, H9 (WA-09), was used as a control.

**Figure 1:**
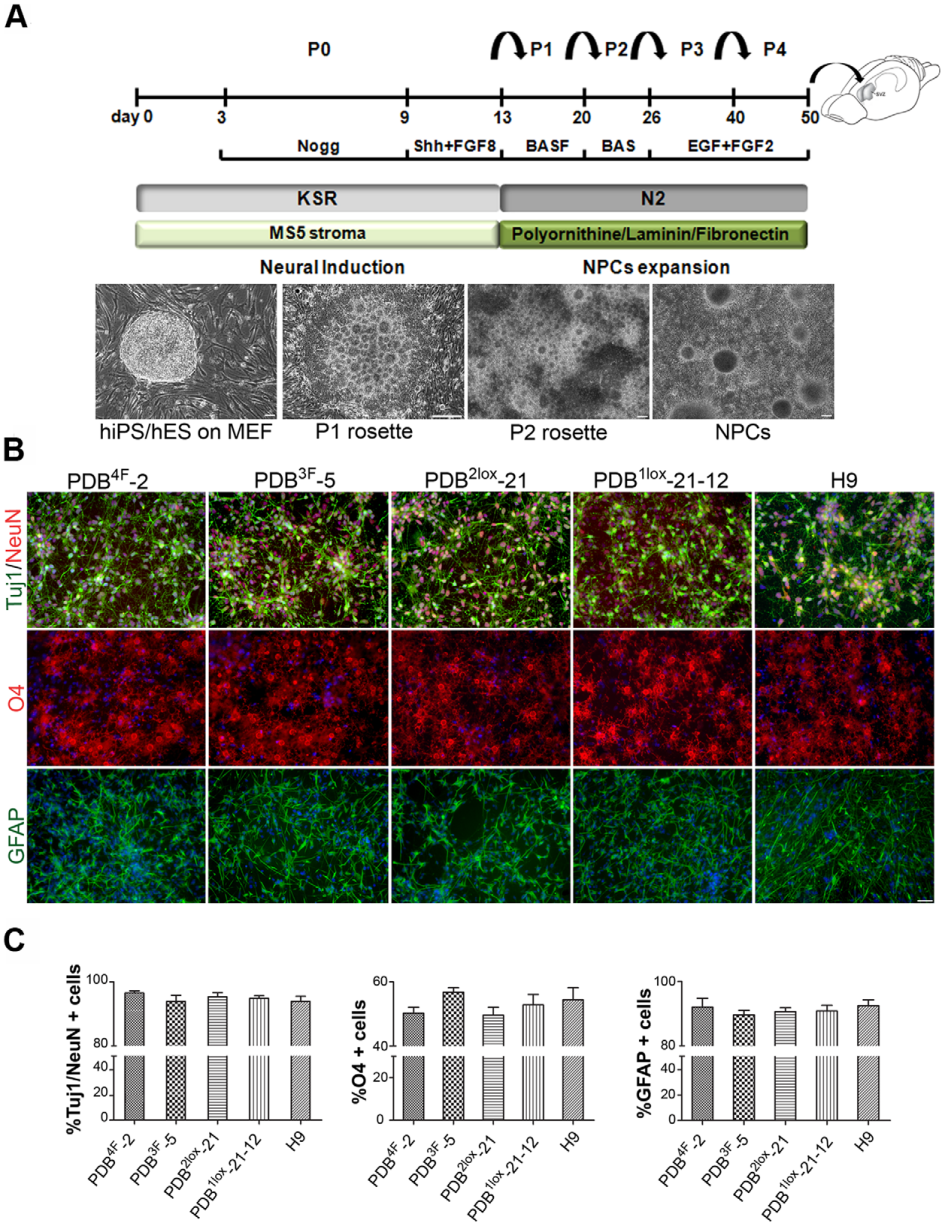
Derivation of neural lineages from hES and iPS cells. (**a**) Schematic representation of the neural differentiation protocol. Phase contrast images of at different stages of neural induction and expansion. (**b**) Immunofluorescence labeling of neurons (TuJ1/NeuN), astrocytes (GFAP) and oligodendrocytes (O4) following differentiation. (**c**) Quantification of neurons, astrocytes, and oligodendrocytes derived from human iPS and ES lines. Scale bars 100 µm in a and 20 µm in b. Errors are SD; n = 3 experiments.

Immunocytochemical analysis of the pluripotency markers showed uniform expression of Nanog, Oct4, Tra1-60 as well SSEA-4 in all hiPSC and H9 lines (**Fig. S1A**). Neural induction and rosette formation was performed under serum-free conditions by coculture on a stromal cell line (MS5) [11] in the presence of Noggin [12]. Rosettes were harvested mechanically on day 10 of differentiation and replated on culture dishes precoated with polyornithine/laminin in N2 media supplemented with growth factors (P1 rosette stage) (**Fig. 1A**). Mechanical isolation of rosettes was repeated after seven days (P2 rosette stage). This process selects for clusters of CNS (Central Nervous System) progenitors and greatly depletes the cultures of neural crest precursors. A second selection is performed 15 days later and neural precursor cells (NPCs) are subsequently cultured and passaged for an additional 3 weeks. At day 50 of differentiation, immunocytochemical analysis reveals that >90% of cells were immunoreactive to nestin and Pax6 (**Fig. S1B**) in all four iPS lines tested as well as the hES control. Immunoreactivity to Ki-67 (44% ±1.7) demonstrated a high proliferative capacity among young neural precursors, comparable across the five lines used (p>0.5). These data suggest that neural induction and neural precursor cell proliferation are not impacted by excision of reprogramming factors or the absence of *c-myc.*

### Differentiation potential along the three neural lineages

Previous studies have assessed the potential of human ES cell and iPS cells to undergo neural and neuronal differentiation in vitro [13] and the differentiation towards neurons expressing dopaminergic markers [14]. Here we extended those studies to address whether neuronal as well as glial lineages can be derived at comparable efficiencies from excised and non-excised iPS cells and from human ES cells. Neuronal differentiation was induced by withdrawal of EGF/FGF2 and exposure to brain-derived neurotrophic factor (BDNF) and ascorbic acid (AA) for three weeks which resulted in a high yield (>90%) of NeuN+/Tuj1+ neurons. Oligodendrocyte differentiation, as assessed by the expression of surface antigen O4 and appropriate morphology, was induced in the presence of BDNF, AA, PDGFR-AA, sonic hedgehog, FGF-8 and cAMP for six weeks following EGF/FGF2 withdrawal. Quantification of glial phenotypes showed that hES as well hiPS derived NPCs were similarly competent and efficient (56.5% ±1.9) in acquiring oligodendrocyte identity (**Fig. 1B, C**). For directed differentiation towards an astrocytic fate, NPCs from each of the lines were differentiated in medium containing 5% FBS, for six additional weeks. Again, equal astrocytic potential across all five tested lines was observed by immunocytochemistry for glial fibrillary acidic protein (GFAP, 91.7% ±1.46) (**Fig. 1B, C**). Thus, neither the number of reprogramming factors used nor their excision impacted in vitro differentiation potential. Furthermore, these hiPS lines exhibited a similar temporal course and capacity for neuro-epithelial, neuronal and glial cell specification, in comparison to human ES cells.

### Tranpslantation in the subventricular zone

We subsequently performed in vivo experiments in order to further define a potential impact of reprogramming strategy on *in vivo* function. We were particularly interested in exploring the *in vivo* differentiation behavior of iPS and hES cell derived neural precursors. While previous studies have assessed the in vivo survival of pre-differentiated dopamine neuron precursors, here we analyze the site- specific in vivo integration and differentiation potential of naïve neural precursor cells. The response to in vivo environmental cues may be a more sensitive parameter to address the impact of transgene excision and to compare the behavior of iPS and ES cell progeny.

**Figure 2:**
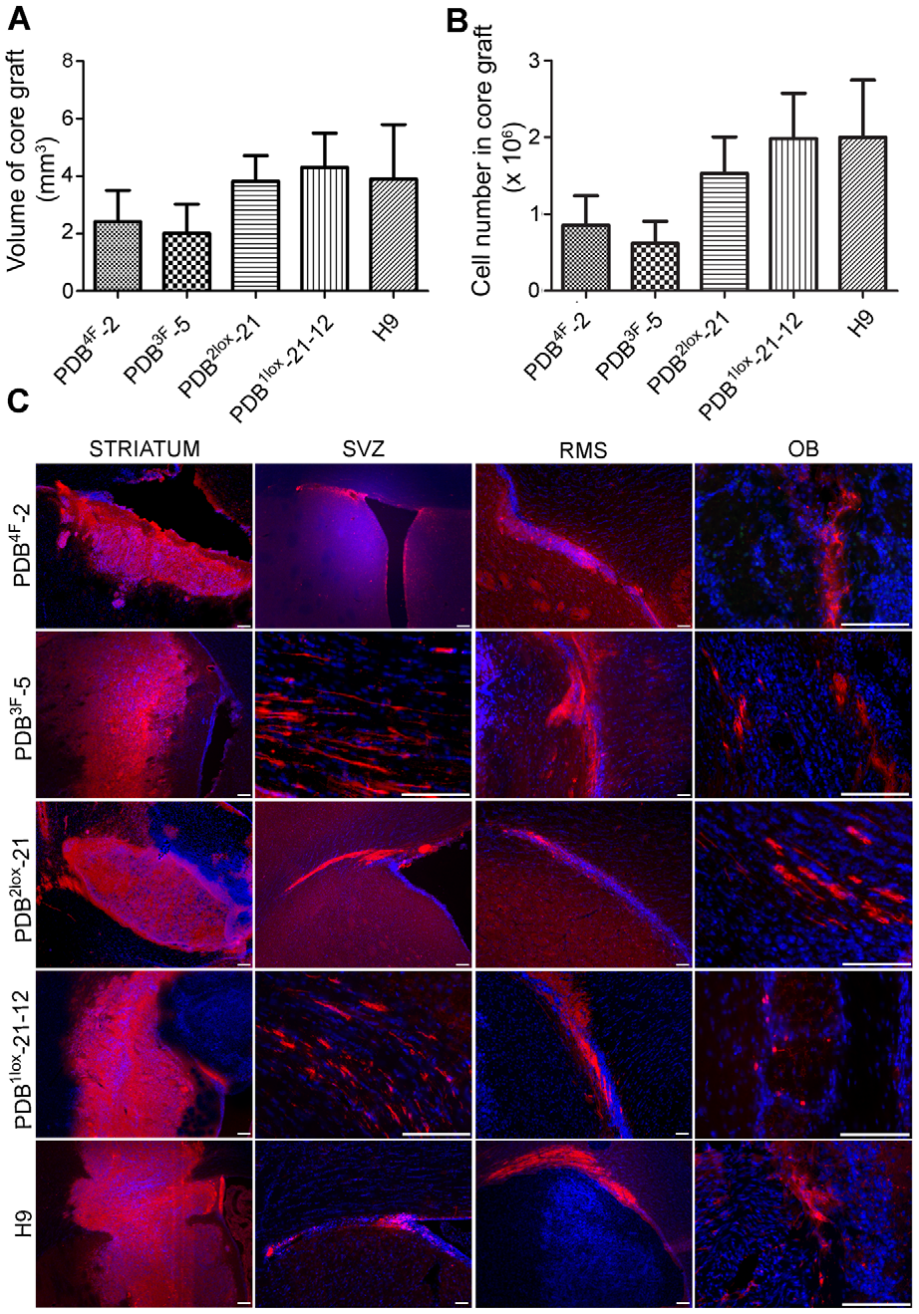
In vivo behavior of hES and hiPS derived neural precursors. (**a**) Graphic representation of the volumes of hiPS- and hES-derived grafts. (**b**) Cell numbers in the core grafts. (**c**) Migratory behavior of transplanted neural precursors (labeled for human nuclear antigen, hNA, in red). Core grafts from all lines are in the striatum while the majority of cells traveled along the rostral migratory stream (RMS) reaching the olfactory bulb (OB). Scale bars, 100 µm. Errors are SD; total animal number = 20.

An identical number of NPCs (day 50 *in vitro*) from each of the hiPS and control lines was stereotactically transplanted into the striatum of adult rats. The animals (n = 20) received a 3-day course of systemic BrdU two weeks post grafting, and were sacrificed at 12 weeks. All surviving animals exhibited a live graft consisting of a core and a large number of migrating cells. Graft volumes as determined by stereological analysis (StereoInvestigator, MBF Bioscience, VT) ranged from 0.95 to 2.8 mm^3^ (**Fig. 2A**). The total cell number within the grafts averaged 1.5 ×10^6^ cells (Optical fractionator, range 0.95–2.5×10^6^ cells) (**Fig. 2B**). One way ANOVA analysis did not demonstrate a statistically significant difference among the volumes and cell counts of the five different groups (p>0.5).

**Figure 3:**
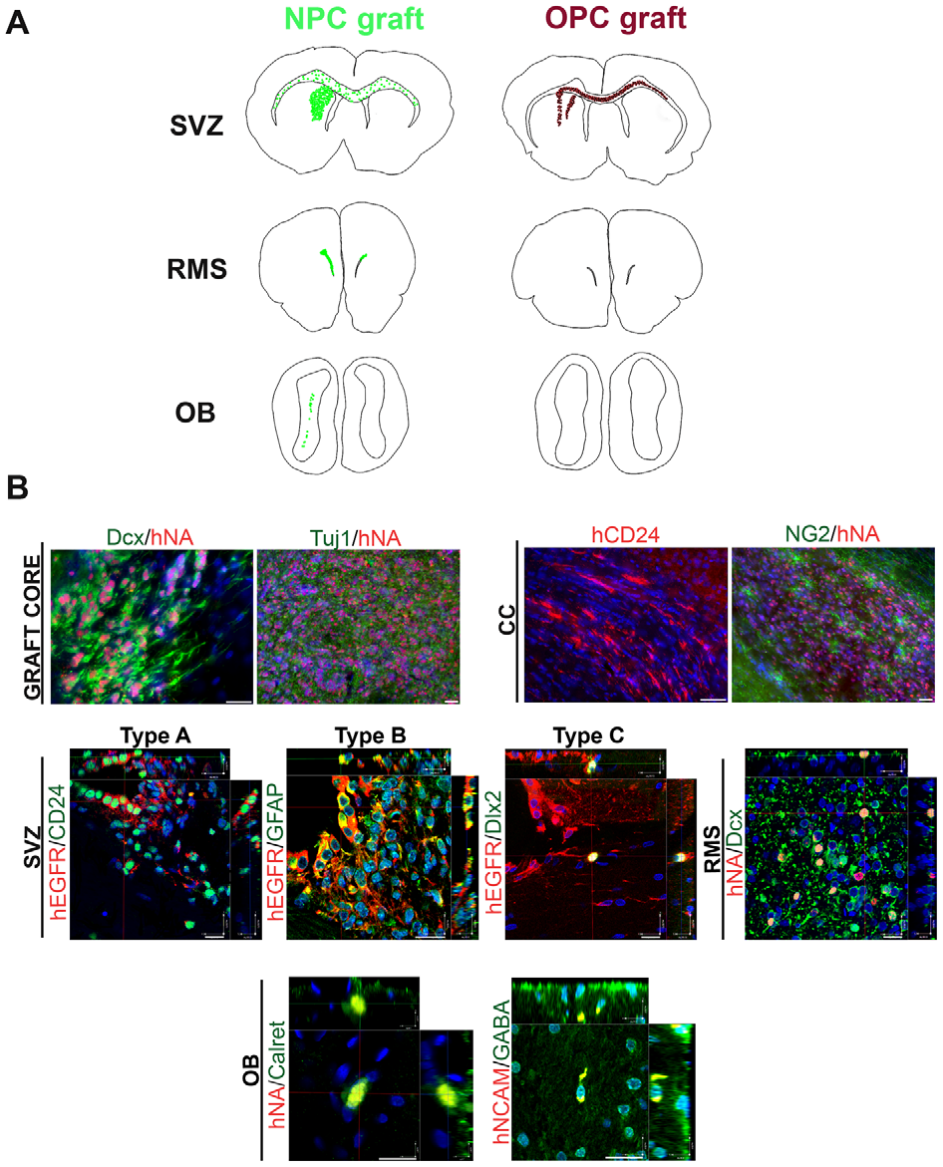
In vivo migration patterns and graft immunohistochemical profiles. (**a**) Representative camera lucida drawings of core grafts and migrating cells, highlighting that OPC grafts were confined to the white matter tracts while NPCs migrated to the RMS and the OB. (**b**) Within the graft cores, cells were predominantly neuronal expressing doublecortin (Dcx; PDB^4F^-2) and TuJ1(PDB^3F^-5). Migrating cells in the corpus callosum were immunopositive for CD24 (PDB^2lox^-21) and a smaller proportion labeled for NG2. In the SVZ, grafted cells acquired region-specific phenotypes: type A cells (EGFR/CD24; PDB^1lox^-21-12), type B cells (EGFR/GFAP; PDB^3F^-5) as well transit amplifying type C cells (EGFR/Dlx2; PDB^2lox^-21). Within the olfactory bulb (OB) rare human cells exhibited an interneuron phenotype, expressing calretinin (PDB^3F^-5) or GABA (PDB^4F^-2). Scale bars, 20 µm. hNCAM  =  human-specific neural cell adhesion molecule; hNA =  human nuclear antigen.

The migration pattern of individual cells was captured by NeuroLucida drawings. Human cells were identified using human nuclear antigen (hNA) or human neural cellular adhesion marker (NCAM) expression. They were distributed ipsi-and contra-laterally in the corpus callosum, but the majority of the cells injected traveled forward along the rostral migratory stream into the olfactory bulb, suggesting a response to local subventricular zone cues (**Fig. 2C**) [15]. There were no differences in migratory patterns among the lines used. To further elucidate the non-random nature of this migratory behavior, we established oligodendrocyte precursor cultures from the H9 line (OPCs, as described above) that were subjected to a FACS sort for O4. O4+ cells were further characterized by the expression of olig2, thus confirming their oligodendroglial identity (**Fig. S2**). The sorted cells were injected at the same coordinates and the animals sacrificed at 12 weeks (n = 6). The migration routes exhibited by the O4 cells were very distinct from those of the naïve neural precursor cells and specifically followed the white matter pathways along the corpus callosum and the internal capsule. In contrast to the neural precursor cell grafts there were only very few cells traveling along the RMS. The cell migration paths are illustrated in representative camera lucida drawings (**Fig. 3A**).

### In vivo phenotypes

The core grafts in all the experimental and control groups were mainly located in the striatum, with small clusters occasionally seen around the corpus callosum. Most of the cells in the core expressed the human markers, human nuclear antigen or human NCAM, were neuronal precursors expressing neuron-specific class III beta-tubulin (Tuj1) as well doublecortin (Dcx)+,(>60%) (**Fig. 3B**, upper panel). A smaller population of glial cells recognized by expression of the NG2 chondroitin sulfate proteoglycan was also observed. GFAP+ human cells were rare and mostly located in the periphery of the core graft (<1%). Host GFAP+ cells were found along the injection site. Human cells that were located away from the core acquired phenotypes typical of the local environment. Human iPS- as well control-derived neural precursors exhibited region specific immunohistochemical profiles, and integrated along the SVZ, giving rise to the three SVZ niche phenotypes [16]. A small proportion of human cells were identified within the ipsilateral and contralateral SVZ where they expressed markers characteristic of the activated type B stem cell population (EGFR+/GFAP+) or type C transit amplifying cells (EGFR+/Dlx2+) [17] (**Fig. 3B**, middle panel). A majority of cells however exhibited a type A or migrating neuroblast phenotype, labeling for CD24, PSA-NCAM and Dcx (**Fig. 3B**, middle panel) as they travelled anteriorly along the RMS. Human cells could be seen at the distal RMS, entering the olfactory bulb, whereby a few cells (<1%) migrated to the granular and periglomerular layers. These cells differentiated into calretenin or calbindin periglomerular interneurons, or γ-aminobutyric acid (GABA) expressing granular interneurons (**Fig. 3B**, lower panel). The number of cells exhibiting interneuron phenotypes was very small (one or two per section) but identical among all hiPS and hES lines used and similar to previously reported data with human ES cells [10] and primary fetal neural progenitors [18]. This region-specific immunohistochemical profile of human cells is identical to that exhibited by endogenous SVZ stem cell progeny and was demonstrated in all 4 hiPS clones and hES line used, as shown in Figure 2 (cell of origin is indicated in the legend). All co-localisations were confirmed by serial 3D reconstruction of confocal sections. No human cells were found in other regions of the olfactory lobe, suggesting that they reached the olfactory bulb selectively by the RMS route rather than by random migration. There was also an absence of neuronal differentiation outside the core graft and the RMS/olfactory area. In contrast the transplanted oligodendrocyte precursors formed a smaller core and exhibited migration along the corpus callosum without any evidence of integration in the SVZ or expression of markers suggestive of SVZ stem cell progeny (**Figure S2**). The OPCs did not reach the olfactory bulb in any of the animals injected.

The immunohistochemical profile of the neural precursors prior to transplantation demonstrates immunoreactivity to nestin and TuJ but not EGFR, Dlx2, and CD24 (**Figure S3**). Similarly the oligodendrocyte progenitors were immunopositive for O4 and olig2 but did not exhibit any SVZ markers. These data suggest a significant cell-autonomous response exhibited specifically by cells at the neural stage in response to environmental cues.

### Proliferation in vivo

**Figure 4:**
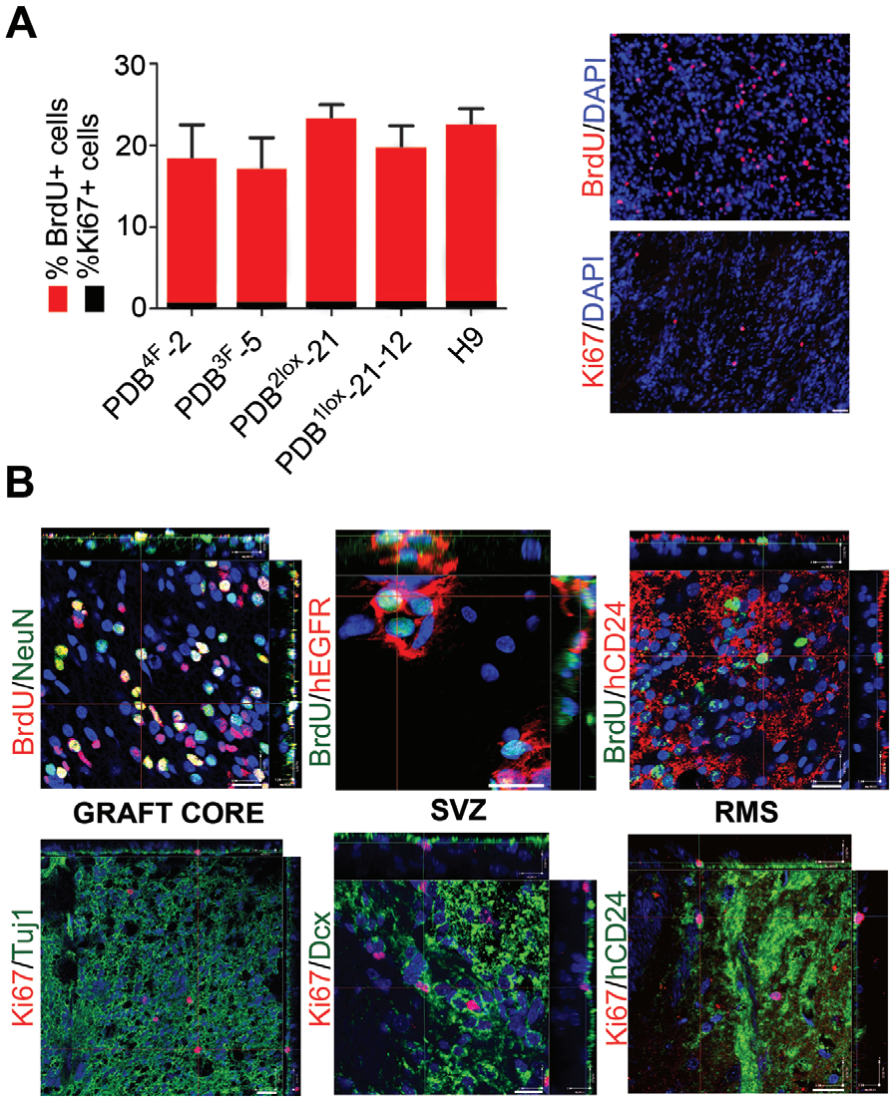
Cell proliferation within the grafts. (**a**) Quantification of BrdU and Ki67 positive cells in the core grafts from hiPS and hES lines. Representative immunohistochemical labeling for BrdU (PDB3F-5) and Ki67 (PDB4F-2). (**b**) BrdU label retention and Ki67 within the graft core, SVZ and RMS. In the core graft BrdU label was mainly found in the neuronal cells (BrdU/NeuN; PDB2lox-21). In the SVZ BrdU was mostly retained by the type B cells (BrdU/EGFR; PDB4F-2) while in the RMS some of the migrating neuroblasts (BrdU/CD24-PDB3F-5) are BrdU positive. Ki67 labeling was very low in all regions. Scale bars, 20 µm. Error bars are SD; n = 20.

The BrdU regimen involved pulse labeling ten weeks prior to sacrifice followed by a chase period for the remainder of the time, allowing identification of label retaining cells and neurons born in vivo. Concomitant labeling with Ki67 allows us to determine the actual rate of proliferation in vivo following graft integration. All five lines tested exhibited BrdU retention and a similar percentage of BrdU+ cells in the graft core (average 19.9% ±6.6; range: 17–23%; calculated stereologically via optical fractionator)[19] (**Fig. 4A**). The predominant phenotype of the BrdU positive cells in the core graft was neuronal (**Fig. 4B**). In the SVZ, the highly proliferative transit amplifying C cells were BrdU negative indicating label dilution. A few human cells residing in the SVZ retained BrdU and co-expressed EGFR, compatible with the slowly dividing type B stem cells. The BrdU label was also retained by the neuroblasts traveling to the RMS (**Fig. 4B**). All grafts from the experimental and control groups exhibited a proliferation capacity of less than 1% as shown by Ki-67 staining (**Fig. 4A**), indicating that most cells stopped dividing 12 weeks post grafting, despite an initial period of proliferation as indicated by the BrdU labels. None of the grafted animal brains harbored a teratoma (absence of alpha-fetoprotein, cytokeratin, myosin) or other type of tumor as assessed by histological analysis on hematoxylin and eosin (H&E) sections.

## Discussion

Reprogramming strategies have evolved rapidly, including the use of micro-RNA [20], non-integrating vectors [21]–[24], mRNA [25], protein [26], [27] or small molecules [28] in lieu of viral transgenes, or microenvironmental modifications to induce endogenous genes [29]. However reprogramming efficiency via most of these methods is extremely low, in the range of 0.001%. The use of integrating vectors offers a relatively more efficient means of reprogramming but also raises safety concerns with the subsequent risk of genetic and epigenetic aberrations [3], [4], the overexpression of proliferation- and potentially neoplasm-associated genes such as c-myc[30] and the incomplete silencing of reprogramming factors following differentiation[5]. Such issues would constitute barriers to the clinical use of iPS progeny [31]. In fact, transmission of iPS clones through the germ line in mice has resulted in tumor formation in 20% of the chimeric progeny, probably due to reactivation of the c-myc transgene [32]. The use of doxycycline-inducible vectors allows greater control over transgene expression but does not eliminate the possibility of residual transgene expression, due to leaky promoters. Thus several groups have introduced integrating vectors that incorporate loxP sites allowing their excision via Cre recombinase once pluripotency is achieved [33]–[35] [9]. Such systems provide the advantages of integrating vectors and higher reprogramming efficiency while allowing for definitive elimination of full transgene expression. Short sequences of vector DNA however remain integrated in the host genome with the potential to interfere with normal function [36].

Despite the rapid evolution of reprogramming strategies, their impact on the potential of iPS cells to stably differentiate along specific lineages has not been extensively studied. Recent literature has reported variable “innate” propensity of ES and iPS lines for preferential differentiation along different lineages [37], as well as interline differences in efficiency of neural induction [13]. Other investigators have suggested that factor-based reprogramming can leave an epigenetic memory of the tissue of origin that may impact directed differentiation [6]. Our data however demonstrate robust neural induction and differentiation into neural and glial lineages, comparable in human ES and iPS lines. This suggests that “innate” differentiation propensity may be related, at least in part, to variations in the specific culture protocols used. A more recent report suggests a similar range of capacity towards motor neuron differentiation [38] in a set of iPS lines derived from 7 different sources, and yet another publication identifies a role for miR-371-3 in specifying the propensity towards neural differentiation [39].

Differentiation along glial lineages is an important tool in disease modeling particularly when non-cell autonomous factors are significant contributors to disease phenotype as is the case in ALS [40], [41], or in cell-based approaches to remyelination. High yield derivation of astrocytes or oligodendrocytes has not been reported to date from human iPS cells. Here, we demonstrate the generation of high percentages of astrocytes (>90%) and oligodendrocytes (>50%) in vitro from all ES and iPS clones presented. The protocols used are based on previously published methods [11], and emphasize the selection of CNS precursors at the expense of neural crest progeny. This is a crucial step in ensuring a higher yield of glial progeny since neural crest derivatives are highly proliferative competitors in culture. However this rate-limiting step relies on mechanical selection based on morphological criteria, and is thus operator-dependent. Ongoing efforts aim to optimize this component of the differentiation protocols, by substituting small molecules or marker-based selection of the cells of interest. Once the cultures are enriched in neural precursors, astrocyte differentiation is achieved via mitogen withdrawal and the addition of serum over several weeks. Oligodendrocyte production is highly dependent upon the elimination of exogenous bFGF and maintaining high concentrations of sonic hedgehog and FGF-8. These protocols exhibit a high reproducibility rate and have in fact led to comparable results among excised and non-excised clones. Remarkably all iPS clones exhibited a profile highly similar to that seen in H9, a well-characterized ES line with a significant neural propensity. Thus reprogramming strategy such as excision of viral transgenes, inclusion of c-myc did not impact in vitro differentiation of human iPS cells along neurons and glia. Obviously this study does not compare multiple lines but is focused specifically on the role of factor excision in lines that exhibit the same background.

The in vivo behavior of iPS cells has not been studied extensively to date. Here we grafted hES and hiPS derived neural progenitors in the adult brain and analyzed their ability to interact with a complex in vivo microenvironment. While the factors that control the cells’ response to cues emanating from the SVZ are not well defined, previous experiments using human fetal neural precursors or hES progeny have demonstrated a remarkable ability for a fraction of cells to integrate that niche. Cells have acquired a neuroblast or a transient amplifying cell phenotype and, very infrequently, a stem cell phenotype that retained BrdU label up to 10 weeks. The human iPS-derived neural precursors also followed the SVZ-olfactory bulb migratory pathway, giving rise to more differentiated neurons and reaching the olfactory glomeruli. Olfactory neurogenesis in the rat is an inefficient phenomenon, with large numbers of cells generated in the SVZ but a very rare percentage incorporating the olfactory glomeruli and differentiating into interneurons. In comparison, human iPS-derived oligodendrocyte precursors followed the white matter along the corpus callosum and did not engage along the RMS or reach the olfactory bulb, suggesting that response to the SVZ niche is in part cell-autonomous. Importantly, the SVZ niche phenotypes exhibited by the iPS derived NPCs were acquired in vivo, likely in response to the local environment.

Other important data points include the absence of excessive proliferation regardless of c-myc status. As expected, NPCs exhibit significant proliferation shortly after transplantation as shown by BrdU retention within the graft core. When examined 12 weeks after grafting, however, proliferation had decreased dramatically as demonstrated by the rare Ki67 expression. More importantly there were no differences in survival, SVZ integration and migration along the RMS and neuronal differentiation among the hiPS lines, all 4 of which behaved similarly to the human ES line. The lines used in this study have also been differentiated into dopamine neurons by Jaenisch and colleagues and transplanted in rat models of Parkinson’s disease with modest survival and some behavioral amelioration [14].

Our data suggest low probability of transgene reactivation or neoplastic transformation via the use of doxycycline-inducible lentiviral vectors to deliver reprogramming transgenes. The inclusion of c-myc in reprogramming did not result in altered proliferative behavior in vitro or in vivo. In addition, Cre-mediated excision did not have a definitive impact on differentiation potential or in vivo behavior of iPS neural progeny. However, more sensitive in vivo paradigms and outcome parameters or longer term studies may be necessary to detect more subtle differences. It is also conceivable that higher levels of transgene expression as in non-inducible vectors may highlight subtle differences among cell lines.

Reprogramming strategies, such as the specific transgenes used, their copy number or their excision had no impact on the ability of hiPS cell progeny to respond to *in vivo* cues and to acquire regional phenotypes within the adult brain. Neither the use of c-myc in reprogramming nor its excision impacted the proliferation rate or graft size of human iPS progeny in vivo. This result cannot rule out that stronger transgene expression would affect in vivo behavior as might be the case with other delivery systems or non-inducible vectors. The use of a single fibroblast source does allow for a clear comparison of the impact of transgenes but future studies will need to address this issue across additional lines.

## Methods

All animal experiments were done in accordance with protocols approved by our Institutional Animal Care and Use Committee (IACUC) and following the National Institutes of Health (NIH) guidelines for animal welfare. The study was also approved by our institutional Ethics committee (ESCRO) under Protocol 2009-06-005.

### Cell culture

Undifferentiated hES (H9 (NIH code: WA09; Wisconsin Alumni Research Foundation, Madison, WI) and hiPS cells (PDB^4F^-2, PDB^3F^-5, PDB^2lox^-21, PDB^1lox^-21-12) were cultured under growth conditions as described previously [42] on mitotically inactivated mouse embryonic fibroblasts (MEFs; GlobalStem, Rockland, MD). The iPS lines were derived from fibroblasts obtained from the Coriell Repository (AG20442; Coriell Institute for Medical Research, Camden, NJ) by Soldner et al [9]. The derivation of the lines as well as the lentiviral vector design and cre-mediated excision are described in detail in Soldner et al. [9]. For neural induction, hES cells were plated on a confluent layer of irradiated stromal cells (MS-5) in knock-out serum replacement (KSR, Invitrogen) medium supplied with Noggin (250 ng/ml). Medium was changed every two days until day 8, when Noggin was replaced with mouse supersonic hedgehog (msSHH, 20 ng/ml) and Fibroblast growth factor (FGF8, 100 ng/ml). All growth factors were purchased from R&D Systems, Minneapolis, MN. Rosette structures were harvested mechanically at day 10 of differentiation (termed Passage 0; P0) and gently replated on 15 µg/ml polyornithine/1 µg/ml Laminin (Po/Lam) coated culture dishes in N2 medium (termed Passage 1; P1). Medium was changed every 2 days, and was supplemented with growth factors as follows: 20 ng/ml msSHH, 100 ng/ml FGF8, 20 ng/ml brain-derived neurotrophic factor (BDNF) (all R&D Systems), and 0.2 mM ascorbic acid (AA)[11] (Sigma–Aldrich, St Louis, MO). After 7 days of P1 culture, rosettes were re-picked and re-plated on Po/Lam coated dishes and kept for an additional week in N2 media containing BDNF, AA and msSHH (termed Passage 2; P2). At the end of the P2 stage, cells were exposed to Ca^2^/Mg^2^-free Hanks balanced salt solution (CMF-HBSS) for 20 min at 25°C and spun at 200 g for 5 min. The cell pellet was gently resuspended in N2 media and plated on PO/lam coated dishes (termed Passage 3, P3). Starting with P3, neural precursor cells (NPCs) were maintained in N2 medium supplemented with 20 ng/ml of fibroblast growth factor 2 (FGF2) and 20 ng/ml of epidermal growth factor (EGF) that was changed every 2 days. As of this stage, the cells will loose rosette formations and form clusters of neural progenitors interspersed among flat cells. The cultures are subsequently passaged at least twice by manual picking of the clusters of central nervous system (CNS) precursor cells. This mechanical purification step ensures the generation of cells highly enriched in CNS precursors and largely devoid of neural crest lineages. Transplanted cells are harvested at this stage.

For neuronal differentiation hES cell-derived CNS NPCs, were subjected to EGF/FGF2 withdrawal at day 50, and cultured for an additional three weeks in N2 media containing BDNF(20 ng/ml) and AA (0.2 mM). For astrocyte differentiation, hES cell-derived NPCs were kept six weeks in N2 media supplemented with 5% Fetal Bovine Serum (FBS, Invitrogen), after mitogen withdrawal. For oligodendrocyte differentiation, after EGF/FGF2 removal, hES cell-derived NPCs were cultured for an additional six weeks in N2 media that contained BDNF (20 ng/ml), AA (0.2 mM), msSHH (100 ng/ml), Fibroblast growth factor (FGF8, 100 ng/ml), Platelet-derived growth factor-AA (PDGF-AA, 20 ng/ml) and dibutyrilic 3′-5′-cyclic adenosine monophosphate, (dbcAMP, 0.2 µM).

For the transplantation of the oligodendrocyte precursor cell (OPCs) population, 100- day old NPCs were dissociated with Accutase (Innovative Cell Technologies, San Diego, CA) and subjected to FACS using O4 antibody (1∶200; Millipore, Billerica, MA) on a MoFlo flow cytometer (Cytomation, Fort Collins, CO).

### Animal surgery

Young adult Sprague Dawley female rats (3 months old at time of grafting, n = 26) were acquired from Taconic and used throughout the study. Stereotactic intracranial transplantation of graft cells was performed under full anesthesia using a mixture of ketamine and xylazine (Hospira, Lake Forest, Illinois/Inc/Ben Venue Laboratories). For all the implants, the animals received a unilateral 1 µl (250,000 cells in sterile HBSS) injection at the following coordinates: striatum: anteroposterior, + 1.6; mediolateral, -1.5; ventral, - 4.2; tooth bar, -2.3. All coordinates relative to bregma and ventral coordinates relative to cortex.

### Immunosuppression

All rats received cyclosporine (Neoral 100 mg/ml; Novartis, Basel, Switzerland) at 20 mg/kg/day intraperitoneally. This regimen was initiated 1 day before grafting and maintained until the animals were sacrificed, 12 weeks following transplantation.

### BrdU administration

The BrdU (Sigma-Aldrich) regimen consisted of two doses of BrdU at 150 mg/kg administered every 12 h for a total of 2 doses over 3 days. This regimen was initiated 2 weeks after transplantation in the SVZ. The animals (n = 26) were sacrificed 10 weeks after the administration of BrdU. BrdU was dissolved in sterile normal saline and .007 M NaOH.

### Tissue processing

Rats were deeply anesthetized with a 25-mg intraperitoneal injection of pentobarbital solution (Nembutal Sodium Solution, Abbott Laboratories Abbott Park, IL). This was followed by transcardial perfusion with 10% Sucrose saline at 4°C (Aldrich-Sigma) followed by 4% paraformaldehyde (PFA) in PBS also at 4°C (pH 7.4). The brains were carefully extracted, post-fixed overnight in 4% PFA at 4°C, and subsequently transferred to 30% sucrose at 4°C until embedding. Optimal cutting temperature compound (O.C.T. Compound, Tissue-Tek, Sakura Finetek, Torrance, CA) was used to embed the brains and sections were cut on a freezing cryostat and stored at −80°C.

### Immunohistochemistry

Sections were washed briefly with PBS/0.1% BSA (Sigma-Aldrich). For fluorescence double immunohistochemistry, sections were first blocked with 10% Normal Goat Serum (Invitrogen, Carlsbad, CA) in PBS and 0.3% Triton X-100 (with the exception of surface antigens, where Triton X-100 was omitted). Some antibodies required a pretreatment step as follows: 30 min in 2N HCl at 25°C for Brdu; 3 min in 100% acetone at −20°C for human nuclear antigen. Primary antibodies were incubated overnight at 4°C. Appropriate secondary antibodies and fluorochromes (Alexa conjugates (Molecular Probes, Invitrogen) were applied for 1 h at 25°C followed by PBS washes, DAPI (Molecular Probes) counterstain and mounted in glycerol. The primary antibodies included: rat anti-Brdu (1∶40, Abcam, Cambridge, UK); Calretinin(1∶2000, Swant, Bellinzona, Switzerland); Calbindin(1∶1000, Swant); EGFR(1∶100; Santa Cruz Biotechnology, Santa Cruz, CA),PSA-NCAM (1∶800, Millipore); Nestin (1∶200 Millipore); CD24 (1∶100, BD Pharmingen, San Diego, CA); Dlx2 (1∶200 Abcam, Cambridge, CA); Tuj-1(Covance 1∶200, Princeton, NJ); human NCAM (Eric-1, 1∶100, Santa Cruz Biotechnology); and human nuclear antigen (1∶50 Chemicon), GFAP(1∶5,000; Dako, Carpineria, CA), NG2 (1∶100; Chemicon), DCX (1∶500 Abcam), O4 (1∶200 Millipore).

### Quantification

Graft volumes were estimated using the Cavalieri estimator probe (StereoInvestigator-6, MBF Bioscience, Williston, VT). Total cell number was assessed in the graft core. Systematic random sampling was applied to the regions of interest (graft core and areas of distribution of human cells) as defined on serial sections. The stereological software was used to design and implement the fractionator probes at a coefficient of error (Gunderson) of ≤0.1.

## Supporting Information

Figure S1 (**a**) Immunocytochemical characterization of hiPS cell lines and H9 line for the expression of the pluripotency markers Tra1-60, Nanog, Oct4 and SSEA-4. (**b**) Immunofluorescence staining of neural precursors derived from four different hiPS clones and H9 line. Neural precursor cells (NPCs) at 50 days in vitro show high expression of Nestin and Pax6. Scale bars, 20μm.
(TIF)

Figure S2 hES or hiPS derived oligodendrocyte precursor cells used for grafting were immunolabeled for the oligodendrocyte cell surface marker O4 (hNCAM/O4) while the markers specific for SVZ type A cells (hNCAM/Dlx2), type B (hNCAM/hEGFR) or type C cells (hNA/Dcx), were absent. O4 cells co-labeled for Olig2. Nuclear counterstain (DAPI) in blue. Scale bar, 20μm.
(TIF)

Figure S3 In vitro immunocytochemistry of hES and hiPS derived neural precursors for phenotypic markers of the SVZ. NPCs (hiPS4) 50 days old were mostly immunoreactive for Nestin and Tuj1 with a low percentage of doublecortin (Dcx) expressing cells. At this stage, neural precursors were negative for markers specific for SVZ type A cells (Dlx2/CD24), type B (hEGFR/GFAP) or SVZ type C cells (hEGFR/Dlx2). Scale bar, 20μm.
(TIF)
