# Supplementary figures and images for: Transgene Excision Has No Impact on In Vivo Integration of Human iPS Derived Neural Precursors
Source: PLoS One. 2011 Sep 22;6(9):e24687. doi: 10.1371/journal.pone.0024687 (PMC3178523; doi:10.1371/journal.pone.0024687)

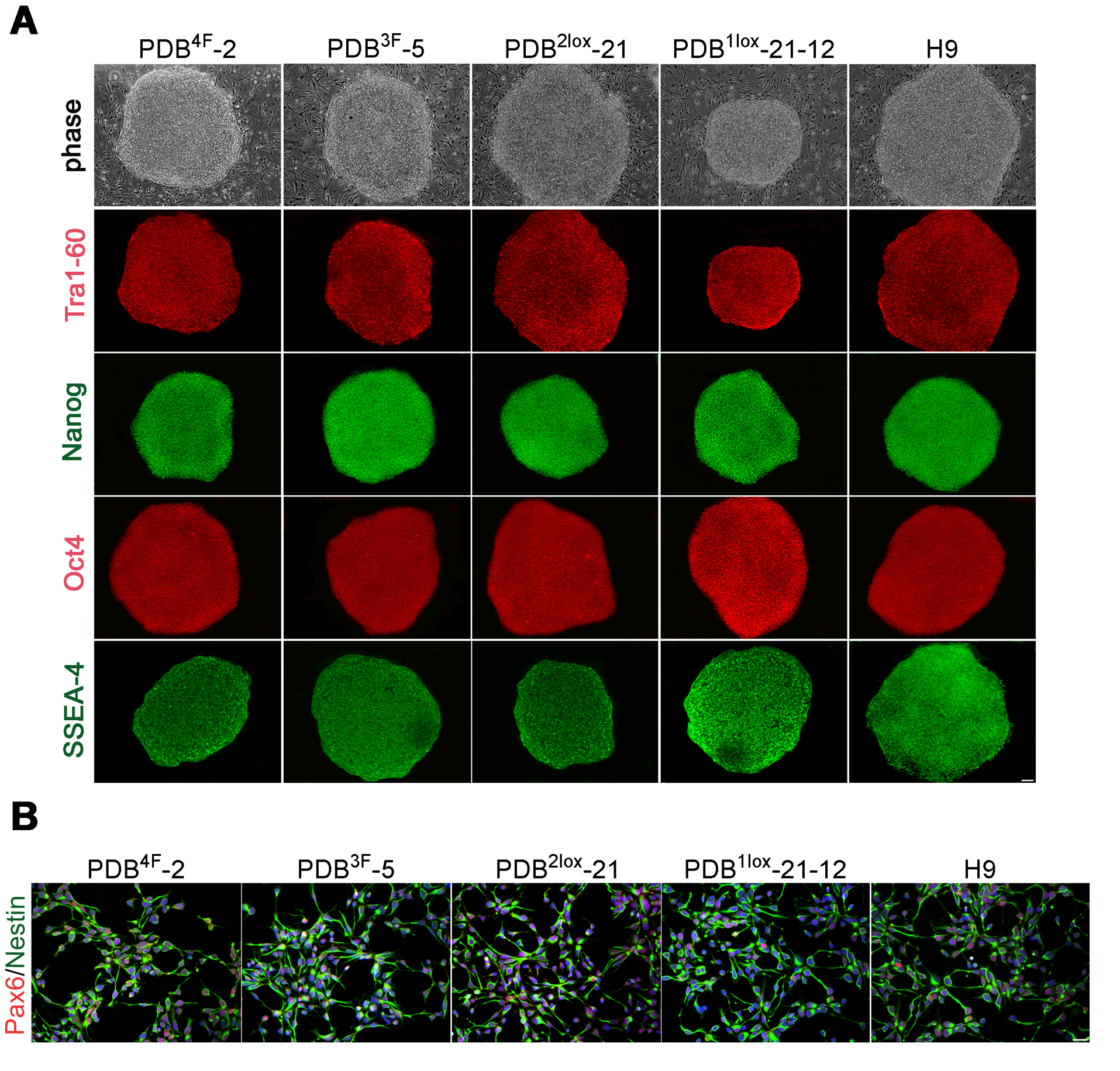

Supplement: Figure S1 — (a) Immunocytochemical characterization of hiPS cell lines and H9 line for the expression of the pluripotency markers Tra1-60, Nanog, Oct4 and SSEA-4. (b) Immunofluorescence staining of neural precursors derived from four different hiPS clones and H9 line. Neural precursor cells (NPCs) at 50 days in vitro show high expression of Nestin and Pax6. Scale bars, 20μm. (TIF) [file pone.0024687.s001.tif]

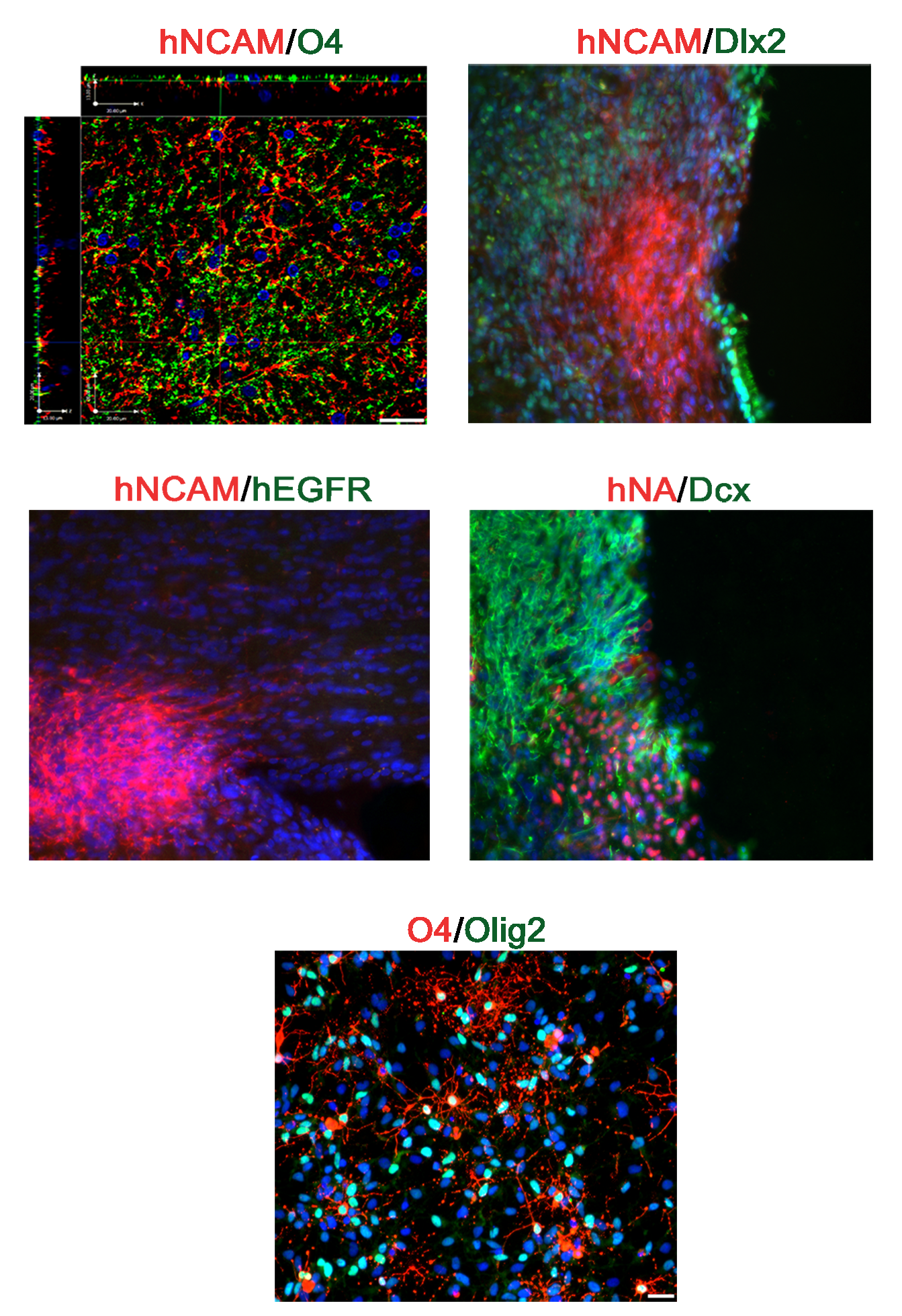

Supplement: Figure S2 — hES or hiPS derived oligodendrocyte precursor cells used for grafting were immunolabeled for the oligodendrocyte cell surface marker O4 (hNCAM/O4) while the markers specific for SVZ type A cells (hNCAM/Dlx2), type B (hNCAM/hEGFR) or type C cells (hNA/Dcx), were absent. O4 cells co-labeled for Olig2. Nuclear counterstain (DAPI) in blue. Scale bar, 20μm. (TIF) [file pone.0024687.s002.tif]

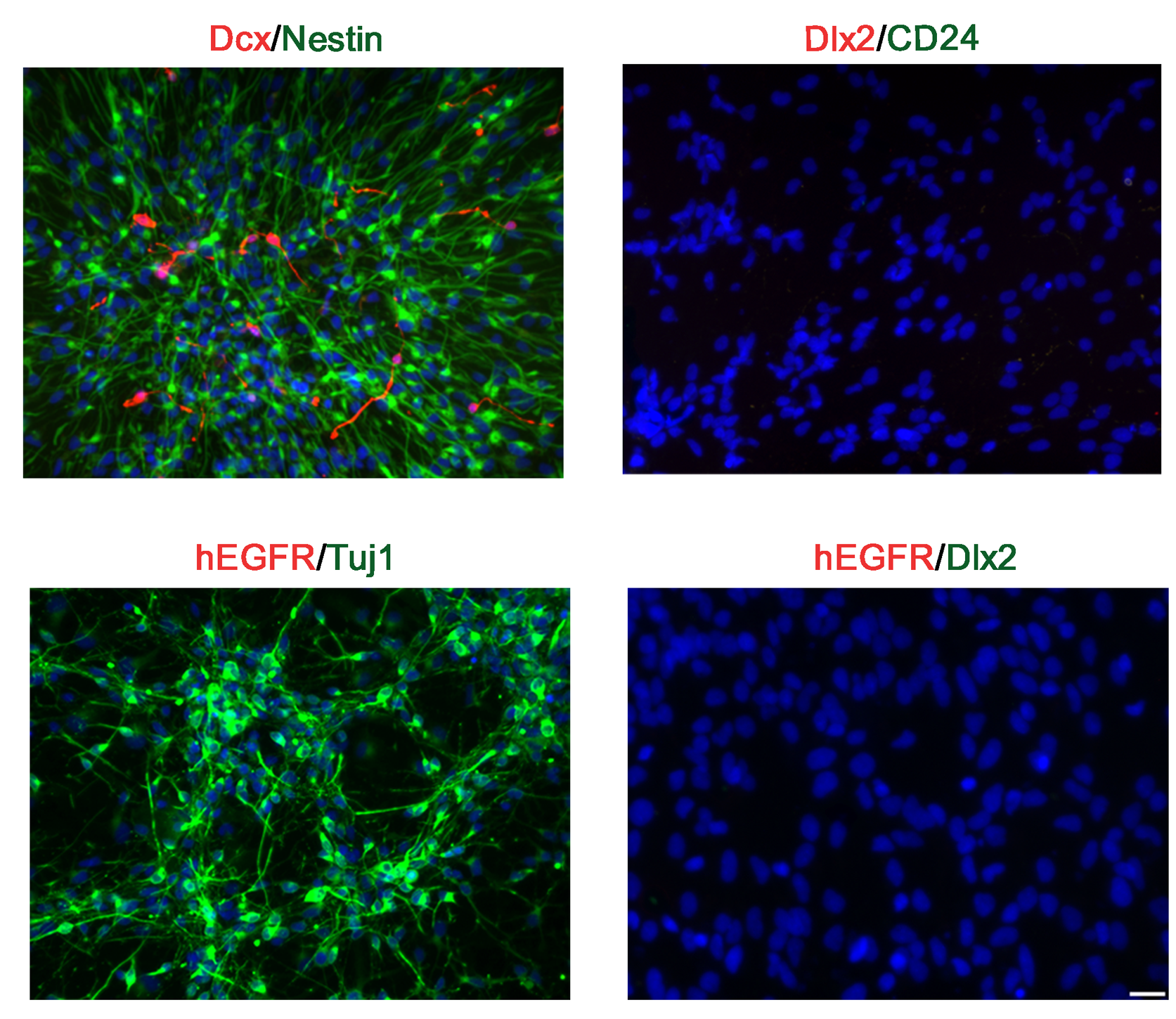

Supplement: Figure S3 — In vitro immunocytochemistry of hES and hiPS derived neural precursors for phenotypic markers of the SVZ. NPCs (hiPS4) 50 days old were mostly immunoreactive for Nestin and Tuj1 with a low percentage of doublecortin (Dcx) expressing cells. At this stage, neural precursors were negative for markers specific for SVZ type A cells (Dlx2/CD24), type B (hEGFR/GFAP) or SVZ type C cells (hEGFR/Dlx2). Scale bar, 20μm. (TIF) [file pone.0024687.s003.tif]
